# Integrative analysis of breast cancer profiles in TCGA by TNBC subgrouping reveals novel microRNA-specific clusters, including miR-17-92a, distinguishing basal-like 1 and basal-like 2 TNBC subtypes

**DOI:** 10.1186/s12885-020-6600-6

**Published:** 2020-02-21

**Authors:** Karel Kalecky, Rebecca Modisette, Samantha Pena, Young-Rae Cho, Joseph Taube

**Affiliations:** 1 Department of Computer Science, Baylor University, Waco, TX USA; 2 Instiute of Biomedical Studies, Baylor University, Waco, TX USA; 3 Department of Biology, Baylor University, Waco, TX USA; 4 Department of Computer and Telecommunication Engineering, Yonsei University, Wonju, South Korea

**Keywords:** Breast cancer, Molecular subtypes, TNBCtype, microRNA, miR-17, INPP4B

## Abstract

**Background:**

The term triple-negative breast cancer (TNBC) is used to describe breast cancers without expression of estrogen receptor, progesterone receptor or HER2 amplification. To advance targeted treatment options for TNBC, it is critical that the subtypes within this classification be described in regard to their characteristic biology and gene expression. The Cancer Genome Atlas (TCGA) dataset provides not only clinical and mRNA expression data but also expression data for microRNAs.

**Results:**

In this study, we applied the Lehmann classifier to TCGA-derived TNBC cases which also contained microRNA expression data and derived subtype-specific microRNA expression patterns. Subsequent analyses integrated known and predicted microRNA-mRNA regulatory nodes as well as patient survival data to identify key networks. Notably, basal-like 1 (BL1) TNBCs were distinguished from basal-like 2 TNBCs through up-regulation of members of the miR-17-92 cluster of microRNAs and suppression of several known miR-17-92 targets including inositol polyphosphate 4-phosphatase type II, INPP4B.

**Conclusions:**

These data demonstrate TNBC subtype-specific microRNA and target mRNA expression which may be applied to future biomarker and therapeutic development studies.

## Background

Breast cancer is a heterogeneous group of diseases, each with characteristic etiologies and optimal treatments. Expression of hormone receptors, estrogen receptor (ER) and progesterone receptor (PR), or human epidermal growth factor receptor 2 (HER2) indicates responsiveness to therapies targeted at these proteins. However, for the approximately 20% of breast cancer patients with tumors negative for such markers, termed triple-negative breast cancer (TNBC), there is presently a lack of effective targeted treatment options [1]. Furthermore, patients with TNBC are presented with worse overall prognoses, necessitating an improved understanding of this disease [2].

Intertumoral heterogeneity within TNBC has been revealed by recent studies [3–5], which show that intrinsic molecular subtyping may be used to separate TNBCs into between four and six subtypes variously labeled as basal-like 1 (BL1), basal-like 2 (BL2), mesenchymal (M), mesenchymal stem-like (MSL), immunomodulatory (IM), and luminal androgen receptor (LAR). Further work has revealed that an abundance of either infiltrating lymphocytes or tumor-associated stromal cells within the sample was the primary determinant specifying the IM or MSL subtype, respectively, resulting in a consensus of four intrinsically-defined TNBC subtypes (BL1, BL2, M and LAR) [4]. Indicating the significant distinctions within TNBC, segregation into these categories yields distinctions in progression with BL1 patients showing significantly greater rates of pathological complete response (pCR) and BL2 patients showing significantly higher rates of distant relapse [4]. Further analysis of the molecular basis for these differences will help to uncover actionable targets to improve outcome.

microRNAs (miRNAs), single-stranded RNA molecules capable of suppressing target gene expression by binding to the 3’UTRs of complementary mRNAs, have emerged as key regulators of cell phenotype and as a potential therapeutic modality in breast cancer [6, 7]. Breast cancer imposes significant disruptions to the expression of many miRNAs and dozens of specific regulatory links between microRNAs and tumor suppressing or oncogenic mRNAs have been identified [7, 8]. In order to explore the molecular determinants separating TNBC subtypes, we conducted an independent analysis of breast cancer datasets with the aim of characterizing microRNAs that significantly contribute to differences in gene expression between TNBC subtypes. Herein we show that 1) BL1, BL2, M, and LAR tumors display individually distinct microRNA expression profiles, 2) the set of predicted microRNA targets corresponds to the set of altered genes between each subtypes and 3) validation in vitro that miRNA, including miR-17-92 cluster members, expression differences predicted between BL1 and BL2 subtypes are validated in a set of breast cancer cell lines, contributing to the distinct expression of known target genes. Overall these results highlight the power of integrated bioinformatics analysis to predict molecular functions associated with disease, pointing the way towards the application of these targets in microRNA-replacement or inhibition therapy to potentially modulate tumor phenotype, with the aim of improving patient outcomes.

## Methods

### Breast cancer data acquisition and TNBC subtyping

Human breast cancer expression data and their demographic information were obtained from NIH NCI Genomic Data Commons public database [9], originally acquired in the scope of TCGA-BRCA program and processed using the same pipeline. Only samples with both mRNA and miRNA expression profiling were considered. Selection of TNBC cases and their classification into TNBC subtypes was adopted from results of 4-subtype schema of Lehmann et al. [4].

### Expression data pre-processing and normalization

All analyses were based on raw expression counts downloaded from Genomic Data Commons database. First, mRNAs/miRNAs entries that were not expressed in at least half of samples of any of the TNBC subtypes were filtered out. Next, the default processing pipeline from R package DESeq2 (v.1.20) [10] was applied to normalize the counts and correct outlying values. This includes a size factor estimation using the standard median ratio method, a dispersion estimation using parametric fitting, expression data fitting using negative binomial generalized linear model with the minimum of 7 replicates for outlier replacement and the lower bound of 0.5 on estimated counts.

### Differential expression analysis

Selected TNBC subtypes were compared using DESeq2 differential expression pipeline, performing two-tailed Wald test of the fitted models using the normal distribution as null distribution. For multiple group comparison, one-way ANOVA test with Tukey’s HSD correction was applied over log2-transformed data. FDR was controlled with Benjamini–Hochberg procedure and comparisons with adjusted *p*-value ≤0.05 were considered as statistically significant. The differences in expression between groups of interest were quantified with log2 fold change. Note that DESeq2 reports shrunken log2 fold change to avoid possible bias in low-expressed entries. Tables with complete results are attached. The most significant differences – with respect to their adjusted *p*-values – are illustrated with heatmaps conveniently exported via MetaboAnalyst (v4.0) [11], using an appropriate size of top RNAs and Ward’s method for hierarchical clustering. Up-regulated and down-regulated mRNAs are shown separately, since a vast majority of all top mRNAs falls into only one of these directions.

### Correlation analysis

Correlation between statistically significantly differentially expressed mRNAs and miRNAs was quantified with Pearson’s product moment correlation coefficient and tested for statistical significance in R programming environment. FDR was controlled with Benjamini–Hochberg procedure and correlation coefficients with adjusted *p*-value ≤0.05 were considered as statistically significant.

### Functional and target analysis

Differentially expressed miRNAs were analyzed with mirPATH (v3.0) [12], miTALOS (v2) [13], and miRNet (v2.0) [14] for target gene pathway enrichment. These multiple tools were used for their application of multiple pathway databases (e.g. KEGG, Gene Ontology, and Reactome) and different target databases (including TarBase, microT-CDS, and TargetScan) encompassing both experimentally validated and computationally predicted targets. Some of these tools allow only a limited number of miRNAs on input, in which case the top miRNAs were selected with respect to their statistical significance. Up-regulated and down-regulated miRNAs were analyzed separately in attempt to distinguish which functional results are a subject of up-regulation and down-regulation. All produced results with *p*-value ≤0.05 are attached.

Top 1000 up-regulated and top 1000 down-regulated mRNAs with respect to their adjusted p-value were analyzed with DAVID functional annotation tool (v6.8) [15] to produce clusters of functional annotations. The default parameters with medium stringency were used, computing over the background of the whole human genome. Again, up-regulated and down-regulated mRNAs were analyzed separately. Clusters with enrichment score ≥ 1 containing at least one annotation with adjusted *p*-value ≤0.05 are listed.

miRNet was further utilized to construct core networks of differentially expressed miRNAs and their targets with highest connectivity, setting up the degree threshold appropriately to obtain a network of a reasonable size.

### Selection of candidate pairs in integrative analysis

MicroRNA-mRNA pairs identified during correlation analysis as significantly correlated were filtered for those with correlation coefficient < − 0.5 and with RNAs differentially expressed between BL1 and BL2 with abs (log2 fold change) > 0.5. Next, candidate pairs checked against microT-CDS (v5.0) [16] and TargetScan (v7.2) [17] target prediction databases with the default parameter settings, selecting pairs present in either database directly or indirectly with a closely related paralogous mRNA. Furthermore, candidate pairs were also narrowed to RNAs, the expression profiles of which showed a possible effect on survival rate of TNBC cases in METABRIC cohort based on visualization by Kaplan-Maier Plotter web tool [18] with trichotomization of samples. Since the low number of TNBC cases is not sufficient to achieve a high statistical power in survival analysis, the RNAs with the largest impact on survival outcome were selected even though the difference might not be statistically significant.

### Cell culture

Cells were obtained from ATCC and cultured according to the provided recommendations: RPMI with 10% fetal bovine serum and 1% penicillin/streptomycin (HCC70) or DMEM with 10% fetal bovine serum and 1% penicillin/streptomycin (MDA MB 468).

### RNA expression

RNA was extracted from cultured cells using Trizol (Invitrogen) according to the manufacturer’s protocol. For detection of microRNA species, purified RNA (250 ng) was subjected to microRNA-specific RT-PCR using the Taqman primer/probe system (Applied Biosystems) and the High-capacity reverse transcription kits (Applied Biosystems) followed by qPCR on the QuantStudio 5 (Applied Biosystems). For detection of mRNA, purified RNA (500 ng) was subjected to reverse transcription using random primers (Applied Biosystems), followed by qPCR using mRNA-specific primers and SYBR Green Universal Master Mix (Applied Biosystems). Expression was quantified using the delta-delta Ct method, normalized to either small nucleolar U6 (microRNAs) or GAPDH (mRNAs) and plotted in reference to the average of all control samples using Prism version 6 (GraphPad Software). Students t-test was used for comparing expression values between two samples.

## Results

### Breast cancer dataset and TNBC subtypes

**Fig. 1:**
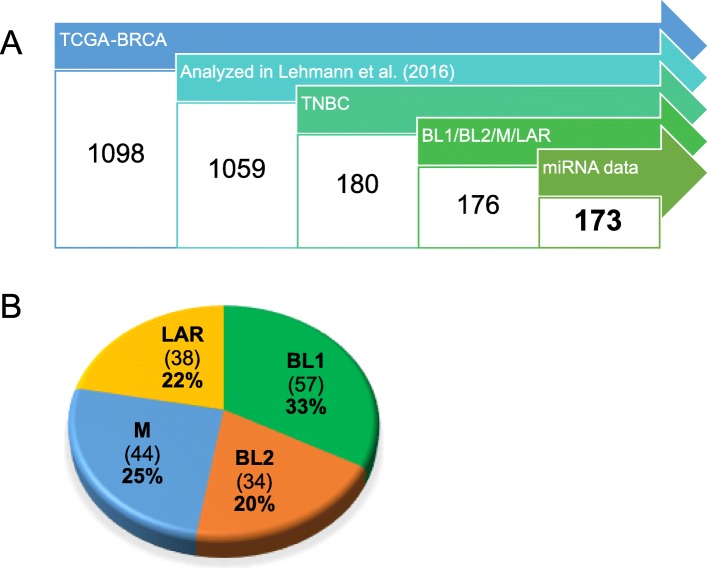
Schematic for selection of cases within TCGA. **a** Data cases from TCGA-BRCA project were filtered for those analyzed and reliably subtyped in Lehmann et al. [4] for those with both mRNA and miRNA expression profiles available. **b** Distribution of TNBC subtypes

The NIH NCI Genomic Data Commons (GDC) database [9] contains mRNA expression profiles of 1098 cases of human breast cancer from TCGA-BRCA project [19]. Lehmann et al. [4] analyzed expression data of 1059 of these cases, identified 180 TNBC cases and 176 of them assigned among the subtypes BL1, BL2, M, and LAR. Adopting this subtyping, we next selected cases for which microRNA expression data were also available, resulting in 173 cases (Fig. 1a; list of case IDs and corresponding subtypes are in Additional file 1) with 60,483 quantified mRNAs and 1881 quantified microRNAs using RNA-Seq and miRNA-Seq technologies. The distribution of individual subtypes is shown in Fig. 1b. These groups are approximately balanced and each of them contains more than 30 samples.

Demographic details for individuals with TNBC grouped by the subtypes are listed in Table 1. All persons are female, approximately one-third black or African American, and predominantly diagnosed with ductal or lobular neoplasms. The most frequent age at diagnosis is in the 40’s although this trend is shifted to the 50’s for BL2 subtype, whereas M and LAR subtypes have a notable proportion of cases diagnosed in the 20’s and 30’s. Based on the monitored vital status, reported mortality for the LAR subtype is almost double the rate for other subtypes.

**Table 1:** Demographic overview

	BL1	BL2	LAR	M
Gender				
Female	100%	100%	100%	100%
Age at Diagnosis				
20–29	2%	3%	13%	5%
30–39	7%	6%	8%	30%
40–49	39%	24%	39%	34%
50–59	21%	41%	21%	20%
60–69	18%	12%	13%	7%
70–79	9%	9%	3%	5%
80+	5%	6%	3%	0%
Ethnicity				
Not Hispanic or Latino	82%	88%	87%	89%
Hispanic or Latino	2%	12%	5%	2%
Not reported	16%	0%	8%	9%
Race				
White	51%	65%	66%	64%
Black/African American	32%	32%	26%	32%
Asian	7%	3%	8%	5%
Not reported	11%	0%	0%	0%
Vital Status				
Alive	89%	85%	71%	86%
Dead	11%	15%	29%	14%
Disease Type				
Ductal or Lobular Neoplasm	100%	85%	97%	86%
Complex Epithelial Neoplasm	0%	15%	0%	7%
Epithelial Neoplasm, NOS	0%	0%	0%	5%
Fibroepithelial Neoplasm	0%	0%	3%	0%
Basal Cell Neoplasm	0%	0%	0%	2%

Distribution of available cases among demographic categories according to TNBC subtypes

### TNBC subtypes express specific patterns of microRNAs

**Fig. 2:**
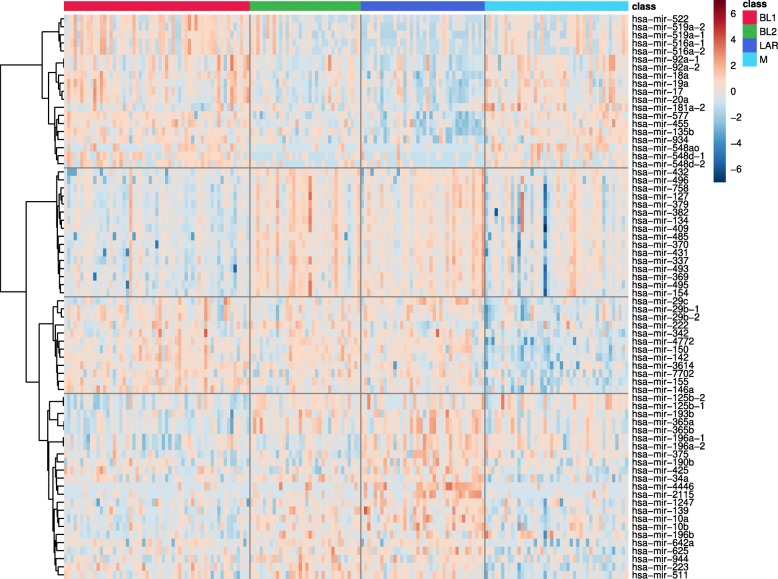
TNBC-subtype specific miRNA expression. Heatmap with expression profiles of top 70 differentially expressed microRNAs across TNBC subtypes. Values are log-transformed and normalized

Exploration of the expression landscape of all TNBC subtypes reveals over 200 microRNAs as differentially expressed with statistical significance. Hierarchical clustering reveals several clusters of 10 or more microRNAs, often with a strong co-expression pattern, that are distinct among the subtypes (Fig. 2). These data support the idea that microRNA expression is tightly linked to intrinsic subtypes within TNBC.

### BL1 and BL2 subtypes shows differential expression in cancer-related groups of genes

**Fig. 3:**
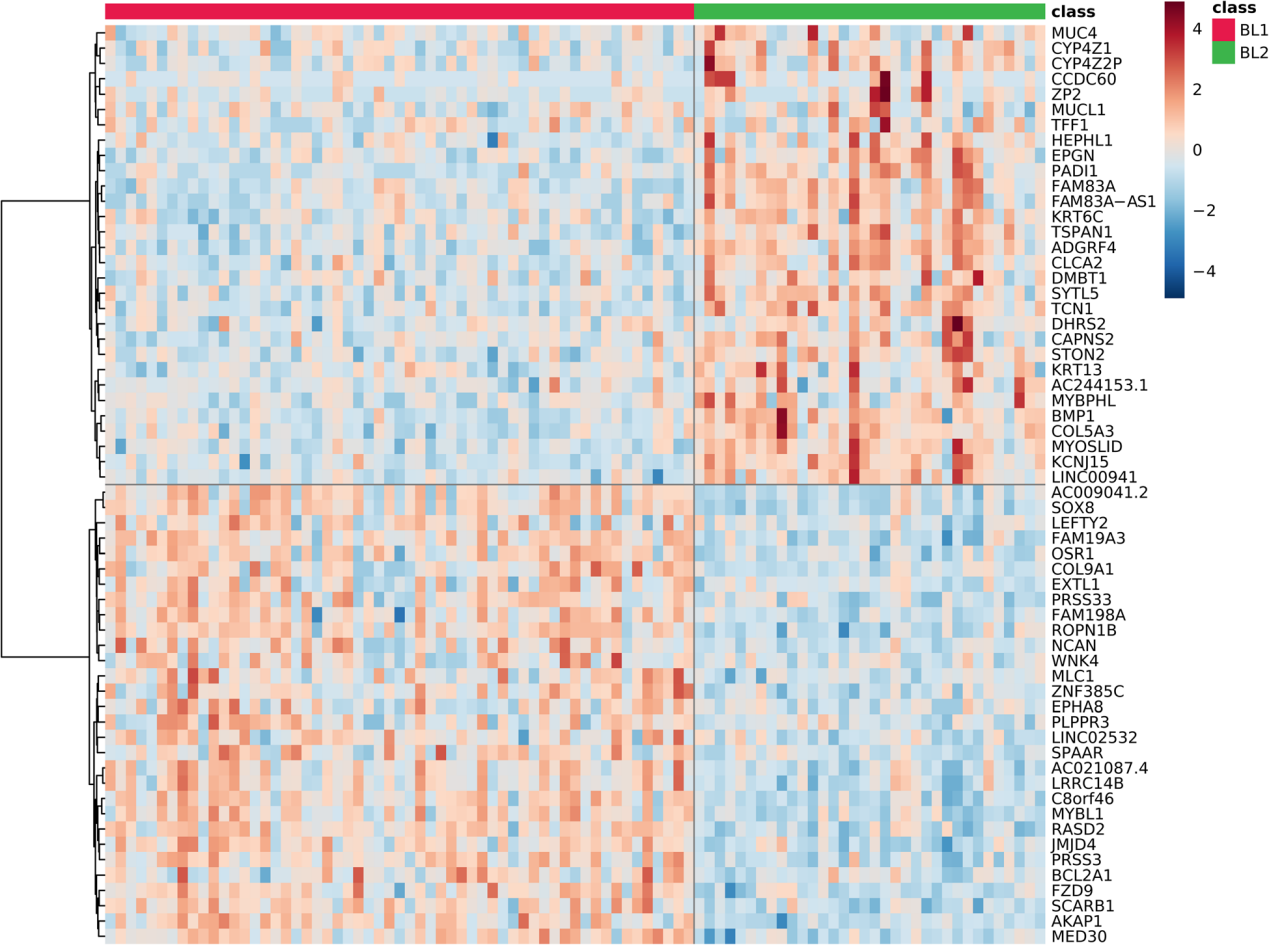
Basal-like 1 and basal-like 2 TNBC-subtype specific mRNA expression. Heatmap with expression profiles of top 60 differentially expressed mRNAs between BL1 and BL2 TNBC subtypes, balanced in each expression direction (30 + 30). Values are log-transformed and normalized

Given the disparity in patient outcomes between BL1 and BL2 [4], we further focused on gene expression signature differences between these subtypes. Differential analysis of gene expression identified over 8000 differentially expressed mRNAs, as shown on a selected example in Fig. 3 (complete list in Additional file 2). Gene ontology analysis of the top mRNAs revealed multiple functional areas relevant to cancer pathology (Table 2, complete list in Additional file 3). Transcripts up-regulated in BL1 are connected with mRNA synthesis and processing, nuclear export, cell division as well as DNA repair and viral processing, whereas transcripts up-regulated in BL2 are related to extracellular matrix, collagen, cell junctions, and cellular membrane components. These differences suggest a role for gene expression in altering interactions with the extracellular environment in BL2, possibly facilitating spread of tumor cells, which would be consistent with more frequent distant relapses clinically observed for the BL2 TNBC subtype [4]. Given the critical nature of these cellular functions, we sought to identify microRNAs with a strong likelihood to regulate mRNA expression differences between BL1 and BL2 subtypes.

**Table 2:** mRNA functional analysis

Down-regulated in BL1 vs BL2	Up-regulated in BL1 vs BL2
Extracellular matrix organization and adhesion	mRNA processing
EGF domains	cell division
Collagen processing and catabolism	nuclear export
Transmembrane components	mitochondria
TSP domains	DNA repair
Cadherin domains	viral processing
Cell junctions	
Leucine-rich repeats	
Fibronectin domains	
Sushi domains	

Most distinct functional areas of mRNAs differentially expressed between BL1 and BL2 subtypes

### BL1 and BL2 subtypes show differential expression in microRNAs targeting cancer-related groups of genes

**Fig. 4:**
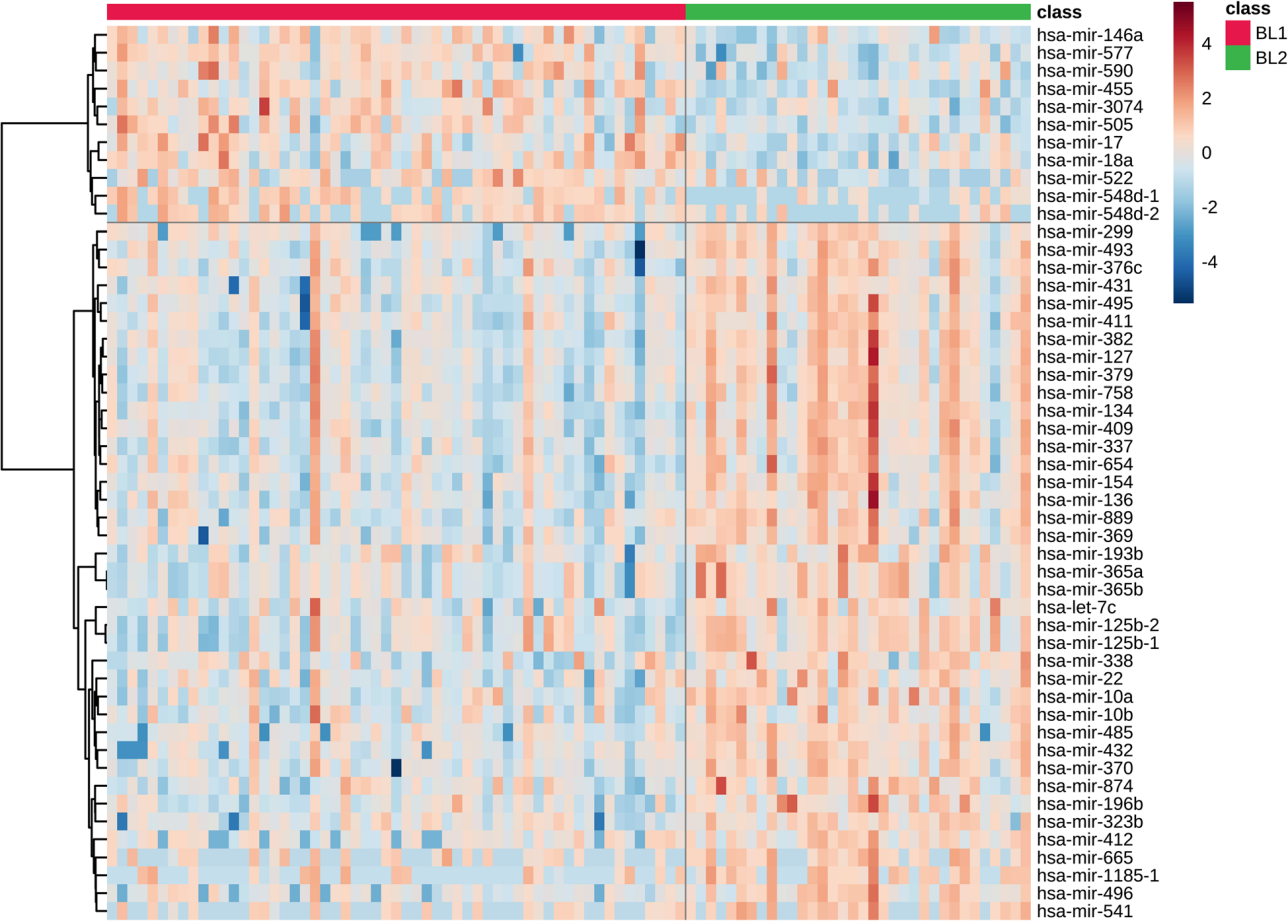
Basal-like 1 and basal-like 2 TNBC-subtype specific miRNA expression. Heatmap with expression profiles of top 50 differentially expressed microRNAs between BL1 and BL2 TNBC subtypes. Values are log-transformed and normalized

**Fig. 5:**
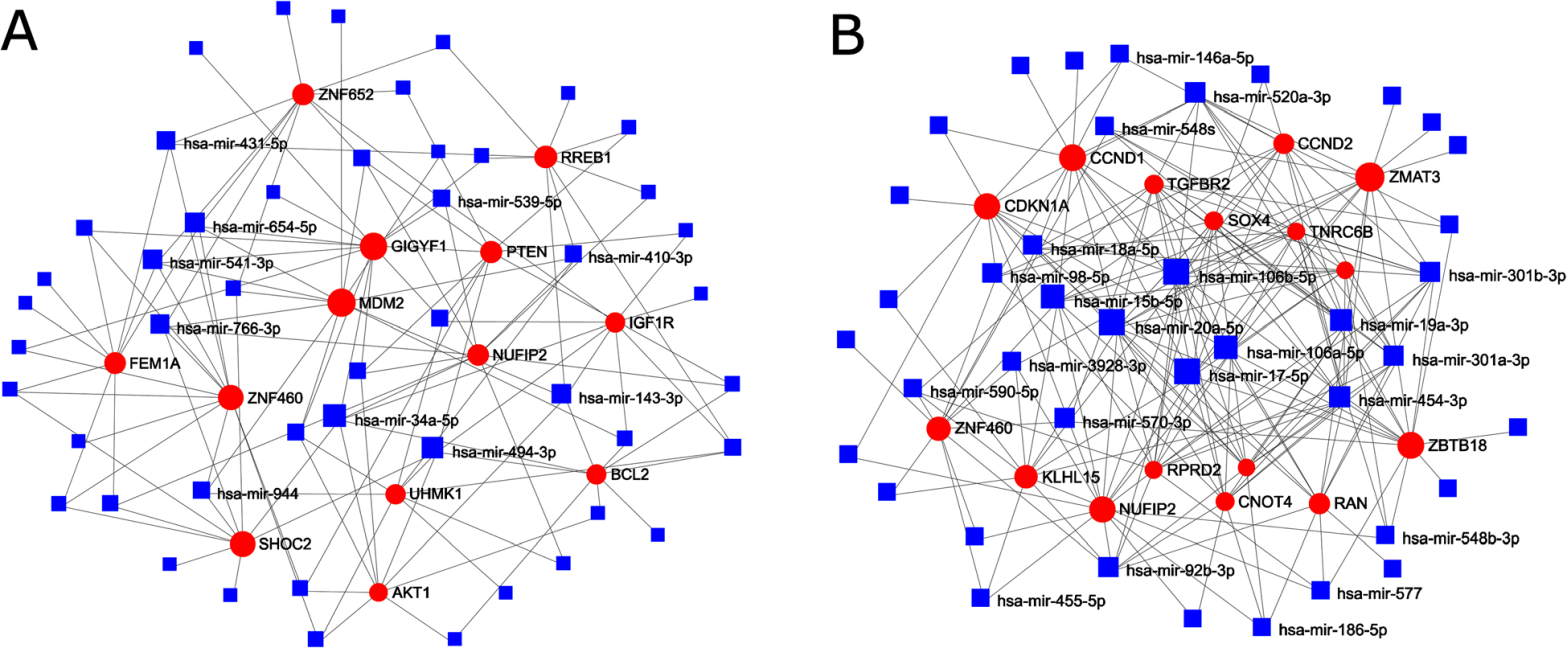
Network of mRNA targets of TNBC-subtype specific miRNA clusters. mRNA-microRNA target networks for differentially expressed up-regulated (**a**) and down-regulated (**b**) microRNAs in BL1 group as compared to BL2 group. The cores for visualization were selected according on node degrees in the graph. The larger the node, the higher node degree

Differential expression analysis identified 159 microRNAs expressed with statistical significance. Top 50 microRNAs are presented in Fig. 4 (complete list in Additional file 4). Subsequent functional analysis of targets of these microRNAs was performed over various gene annotation databases and microRNA target databases, encompassing databases for experimentally validated targets as well as algorithmically predicted targets. In general, many biological functions, each with hundreds of mRNAs differentially expressed, were predicted to be targeted by several dozen microRNAs (Additional file 5). The detected functions are often cancer-related, but also extend to many other biological processes, and frequently are linked to both up-regulated and down-regulated microRNAs, illustrating the regulatory complexity of microRNAs. Although these results do not identify any particular microRNA-mRNA pairs relevant for BL1 and BL2 subtype distinction, it affirms the role of microRNAs in the etiology of the subtypes. Separate network analysis of differentially expressed up-regulated and down-regulated microRNAs and their targets confirms that mRNA targets in the network interaction core are strongly linked to cancer biology, including functions such as cell growth and cell cycle, apoptosis regulation, vasodilation, glucose metabolism, and inflammation (Fig. 5).

### Integrating differential expression, correlation, target and survival analysis identifies candidate microRNA-mRNA pairs relevant to BL1 and BL2 subtype distinction

**Fig. 6:**
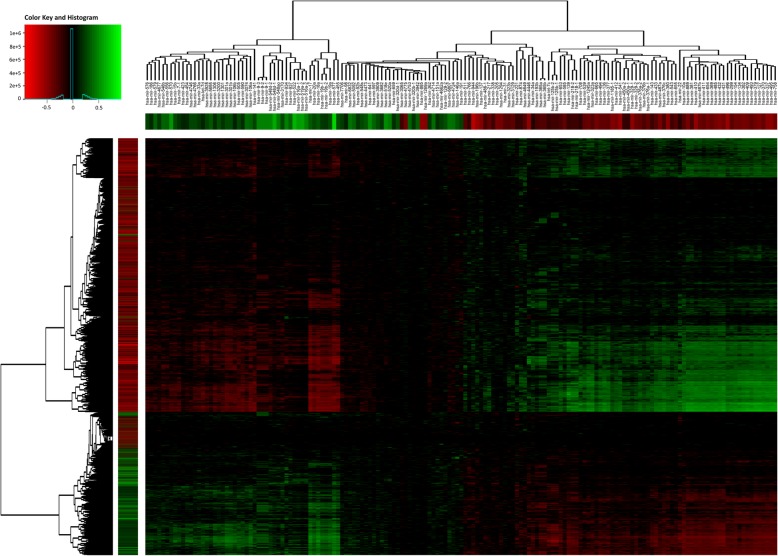
Correlation between BL1 and BL2 differentially expressed miRNAs and mRNAs. Heatmap with Pearson’s coefficients of correlation between expression profiles of differentially expressed mRNAs and microRNAs. Side bars denote log2 fold change of respective RNAs; green – positive log fold change (BL1 vs BL2)

In order to identify nodes likely to underlie biological differences between BL1 and BL2 tumors we conducted network analysis, combining predicted miRNA-mRNA pairs with BL1-BL2 differential expression data. Further, we sought to find suitable pairs of microRNAs and their targets for experimental validation of their expression and regulation in BL1 and BL2 TNBC cell lines. Expression patterns of microRNAs should exhibit significant anti-correlational tendency with the expression levels of their targeted mRNAs. Therefore, we compared expression profiles of all differentially expressed RNAs and all significantly non-zero correlations were selected as outlined in Fig. 6 (complete table with values in Additional file 6).

**Fig. 7:**
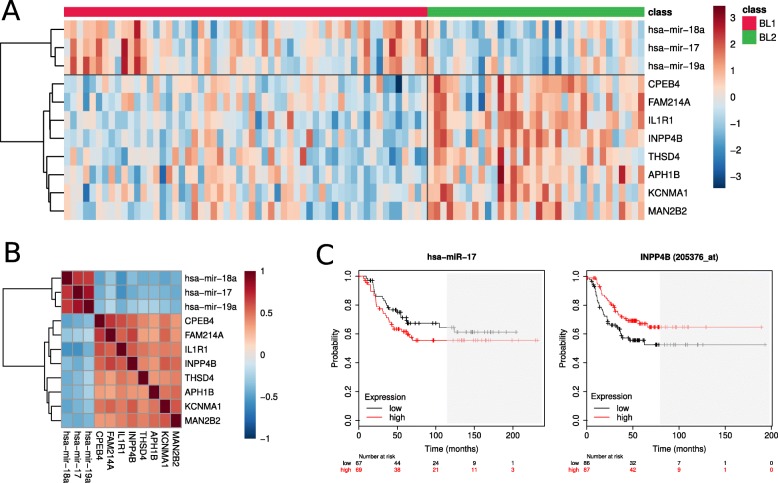
Expression profiles and correlation of selected mRNAs and microRNAs. Heatmap with expression profiles in BL1 and BL2 (**a**) and their Pearson’s correlation coefficients (**b**) of mRNAs and microRNAs selected in integrative analysis. Expression values were log-transformed and normalized. **c** Example of survival plots of selected RNAs with trichotomization of samples according to the expression. Areas with a low number of remaining samples (< 20) are shaded

To identify mRNA-miRNA pairs likely to exhibit a biological relationship, we considered only pairs with correlation coefficient below − 0.5, consisting of RNAs with absolute log2 fold change above 0.5. As a result, 280 candidate pairs remained, consisting of 27 unique microRNAs and 168 unique mRNAs. To refine our selection, we chose only pairs identified by target prediction databases and further, only considered mRNAs with a possible impact on survival outcomes, resulting in 10 candidate pairs of 3 unique microRNAs and 8 unique mRNAs (Table 3). Their correlations and a heatmap of expression within BL1 and BL2 TNBC subgroups are shown in Fig. 7, as well as an example of survival charts.

**Table 3:** Selected candidate mRNA-microRNA pairs

microRNA	mRNA	Correlation		Log2 Fold Change B1 vs B2		TNBC Pro-Survival Direction		Predicted Binding	
		PCC	adj. p-value	miRNA	mRNA	miRNA	mRNA	Orientation	mRNA
hsa-mir-17	IL1R1	−0.56	1.17e-12	1.04	−1.09	Down	UP	3p	IL1R1
hsa-mir-17	INPP4B	−0.55	4.21e-12	1.04	−1.31	Down	UP	3p	INPP4B
hsa-mir-18a	APH1B	−0.52	1.46e-10	0.98	−0.55	Down	UP	3p	(APH1A)
hsa-mir-18a	CPEB4	−0.50	6.02e-10	0.98	−0.83	Down	UP	3p	(CPEB1/3)
hsa-mir-18a	FAM214A	−0.52	1.00e-10	0.98	−0.54	Down	UP	3p	(FAM214B)
hsa-mir-18a	INPP4B	−0.52	1.62e-10	0.98	−1.31	Down	UP	3p	INPP4B
hsa-mir-18a	KCNMA1	−0.50	6.79e-10	0.98	−1.04	Down	UP	5p	KCNMA1
hsa-mir-18a	MAN2B2	−0.54	1.17e-11	0.98	−0.52	Down	UP	3p	(MAN2B1)
hsa-mir-18a	THSD4	−0.52	7.80e-11	0.98	−0.92	Down	UP	3p	THSD4
hsa-mir-19a	IL1R1	−0.52	9.24e-11	0.87	−1.09	Down	UP	3p + 5p	IL1R1

mRNA-microRNA pairs computed by the integrative analysis combining correlation, differential expression, survival and target analysis. *PCC* Pearson correlation coefficient. Target mRNAs in parenthesis are paralogs of the investigated mRNAs

### Predicted difference in miRNA and target expression is recapitulated in breast cancer cell lines

**Fig. 8:**
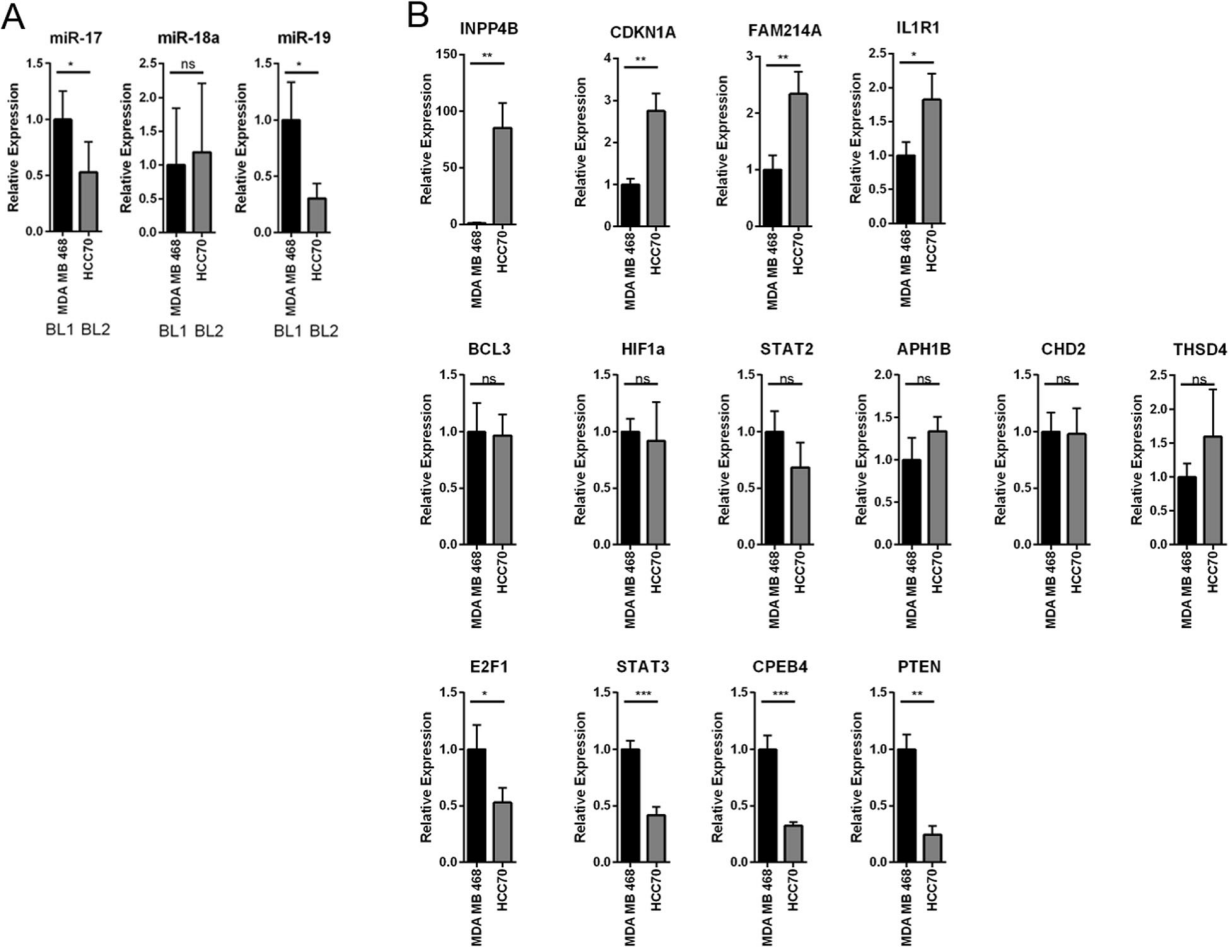
miR-17 and miR-19a and targets are differentially expressed between BL1 and BL2. **a** Expression of miRNAs was determined in the indicated cell lines via miR-specific qPCR. **b** Expression of mRNAs was determined in the indicated cell lines via qPCR. Values are normalized to the mean of three replicates for MDA-MB-468. The mean and standard deviation of three replicates are plotted. Student’s t-test was applied to determine statistical significance between MDA-MB-468 and HCC70

We next sought to validate the predicted expression differences of microRNAs and their targets that were shown to be distinct between the BL1, BL2, and M subtypes of TNBC, as recapitulated in breast cancer cell lines. For this, we chose cells lines previously identified as corresponding to specific TNBC subtypes (HCC70 = basal-like 1; MDA-MB-468 = basal-like2; and MDA-MB-231, SUM159 and Hs578t = M) [3]. We focused on the network of miRNAs and mRNAs identified as distinct between BL1 and BL2 tumors (Fig. 5b, Table 3). Expression of miR-17 and miR-19a was elevated in MDA-MB-468 (BL1) cells as compared to HCC70 (BL2) cells while miR-18a was not statistically significant (Fig. 8a). miR-17, miR-18a, and miR-19a are co-expressed from the MIR17–92a cluster of microRNAs and are predicted to target mRNAs regulating cell cycle, apoptosis, and signal transduction (Fig. 5 and Table 3). We examined the expression of these predicted targets in HCC70 and MDA-MB-468 cells as representative of the BL1 and BL2 TNBC subtypes. Intriguingly, of the fourteen miR-17-, miR-18a-, and miR-19a- targets tested, only four showed elevated expression in HCC70 (BL2) cells compared to MDA-MB-468 (BL1) cells. Remarkably however, predicted targets of miR-17 and miR-19a, IL1R1 and INPP4B (Table 3), were expressed more strongly in HCC70 (BL2) cells, while the predicted targets of miR-18a were not differentially expressed (Fig. 8b). Thus, TNBC cell lines showed similar anti-correlation between miRNA (miR-17, miR-19a) and mRNA target (IL1R1, INPP4B) as the TCGA-based segregation of TNBC tumors into BL1 and BL2 subtypes (Table 3). In addition, CDKN1A (miR-17 target that did not anti-correlate in the TCGA data) and FAM214A (miR-18a target) also showed elevated expression in the HCC70 (BL2) cells (Fig. 8b).

## Discussion

The significance of microRNAs in cancer cell regulation is still a widely unexplored area. The Genomic Data Commons database is a monumental collection of genetic data for cancer research, encompassing The Cancer Genome Atlas (TCGA) and other projects, creating an opportunity for revealing new microRNA-mRNA pairs impacting cell proliferation. Indeed, there have been attempts to build tools that could, to a certain degree, automatize the search and were applied to TCGA datasets [20, 21]. However, identification of the candidate pairs is a challenging task due to the regulatory complexity and inter-dependence of mRNAs and microRNAs and performing only correlation analysis between differentially expressed mRNAs and microRNAs followed by a network analysis might not be a satisfactory approach. Expression analysis frequently produces thousands of differentially expressed mRNA and correlation analysis yields tens of thousands of candidate pairs. The constructed network then can be unfeasibly large while reducing the network to its most dense core can omit important parts. It is worthwhile to note that the mRNA-microRNA pairs of therapeutic interest are not necessarily the most differentially expressed ones or the ones with highest anti-correlation or the ones in the center of the target network. Reducing the number of candidate pairs based on these criteria solely may not be revealing.

**Fig. 9:**
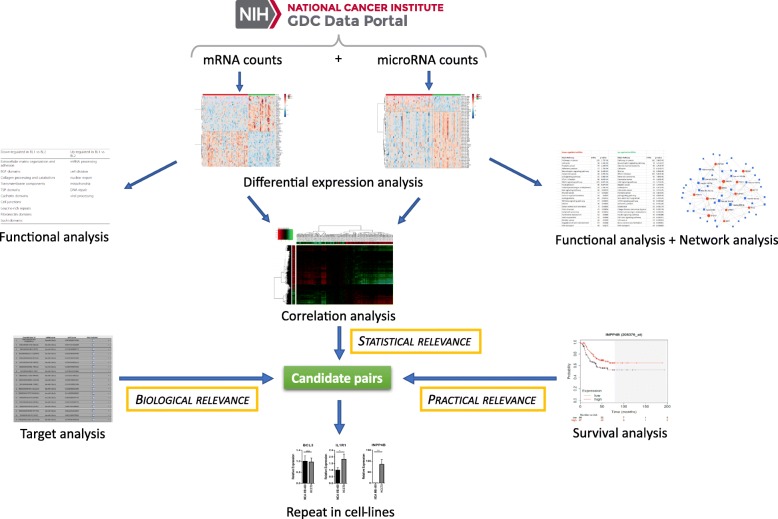
Integrative analysis approach. Raw RNA counts from GDC database were processed in differential expression analysis. Differentially expressed RNAs were further inspected via functional analysis and network analysis (for microRNAs) to confirm that the significant differences are cancer-related. Afterwards, correlation analysis, target analysis and survival analysis were jointly applied to the differentially expressed RNAs to select the best candidates that could affect the difference between BL1 and BL2 subtypes and their outcomes. The candidates were then verified in BL1 and BL2 cell lines

In this study, we have combined correlation analysis and target analysis together with survival analysis, thus integrating statistical and biological relevance with practical relevance (see Fig. 9 for the analytical pipeline). This approach allowed us to perform the final selection of candidate pairs based on less stringent thresholds in each factor while still achieving a reasonable count of the candidates, which are additionally interesting from the therapeutic perspective for their possible impact on survival rates. A very recent publication analyzing TCGA data [22] also performs survival analysis for selection of candidate mRNA-microRNA pairs although differentially expressed mRNAs were pre-filtered and only around 1% of statistically significant ones were analyzed.

Applying the described approach, we have analyzed publicly available triple-negative breast cancer expression data from the GDC database, subtyped into basal-like 1, basal-like 2, luminal androgen-enriched, and mesenchymal cases, where we have focused on differences between BL1 and BL2 groups. Notably, we have found pairs involving several members of the miR-17-92a cluster as more abundantly expressed in BL1 tumors. Importantly, restricting our analysis to TNBC tumors revealed this association which was not apparent in a similar study analyzing all breast cancer cases [23]. Using representative breast cancer cell lines, we also demonstrated elevated expression of miR-17 and miR-19a in BL1, coincident with suppressed expression of CDKN1A, FAM214A, and INPP4B, validating the patient-derived association.

The miR-17-92 cluster, located in an intron of MIR17HG, encodes miRs-17, −18a, −19a, −20a, −19b and -92a. These microRNAs are frequently upregulated in breast cancer [24] and suppress growth control proteins such as E2F1 [25] and PTEN [26]. Despite a predominant view of these miRNAs as oncogenic, several lines of evidence complicate their role in cancer progression. The miR-17-92 cluster is deleted in 21.9% of breast cancer [27] and forced overexpression of miR-17 in breast cancer cell lines reduces their proliferative capacity [28]. Furthermore, the miR-17-92 cluster is suppressed in cancer stem cells (CSCs) in a pancreatic cancer model, facilitating persistent quiescence of this population [29]. Thus, the cellular context is paramount in dictating the function of miRNAs, including miR-17-92.

We observed a consistent anti-correlation pattern between miR-17, miR-19a and Inositol polyphosphate 4-phosphatase II (INPP4B), an inhibitor of PI3 kinase signaling. Indeed, negativity for INPP4B has been identified as a marker for basal-like breast cancer with protein loss in 84% of basal-like breast cancers and loss-of-heterozygosity in 55% of triple-negative, basal-like cancers [30, 31]. Its function as a tumor suppressor was shown through decreased proliferation and Akt activation upon restoration of INPP4B expression in the ER-negative breast cancer cell line, MDA-MB-231 [31, 32]. Consistent with these reports, we observed a lack of INPP4B expression in triple negative, BL1, MDA-MB-468 cells. However, the triple-negative, BL2, cell line HCC70 expressed detectable INPP4 mRNA. In the analyzed TCGA dataset, copy-number variation and mutation data are available only for a fraction of TNBC cases, affecting around 30% cases and suggesting no differences between BL1 and BL2 subtypes.

## Conclusions

Triple-negative breast cancer is a heterogenous disease. Refining the biological distinctions among subtypes within TNBC is critical for improving prognostic information and therapeutic opportunities for patients with these diseases. Here we show that TNBC subtypes express distinct microRNA profiles which are linked to cancer-associated mRNAs. In particular basal-like 1 and basal-like 2 tumors show distinct expression patterns of miR-17-92 cluster microRNAs and targets.

## Supplementary information

**Additional file 1.** Cases - List of used cases from GDC database and their TNBC subtypes.

**Additional file 2.** DE mRNAs - List of all differentially expressed mRNAs between BL1 and BL2 subtypes.

**Additional file 3.** FA mRNAs – Detailed results of functional analysis of differentially expressed mRNAs between BL1 and BL2 subtypes.

**Additional file 4.** DE microRNAs - List of all differentially expressed microRNAs between BL1 and BL2 subtypes.

**Additional file 5.** FA microRNAs – Detailed results of functional analysis of differentially expressed microRNAs between BL1 and BL2 subtypes.

**Additional file 6.** COR – List of all correlated mRNA-microRNA pairs differentially expressed between BL1 and BL2 subtypes.

## Data Availability

Data analyzed in this study are publicly available in NIH NCI GDC data repository (portal.gdc.cancer.gov) and can be accessed with IDs listed in Additional file 1.

## Abbreviations

ANOVA: Analysis of variance
BL1: Basal-like 1
BL2: Basal-like 2
EGF: Epidermal growth factor
ER: Estrogen receptor
IM: Immunomodulatory
LAR: Luminal, androgen-enriched
miRNA: microRNA
MSL: Mesenchymal, stem-like
pCR: Pathological complete response
PR: Progesterone receptor
qPCR: Quantitative Polymerase Chain Reaction
TCGA: The Cancer Genome Atlas
TCGA-BRCA: The Cancer Genome Atlas-Breast Cancer
TNBC: Triple Negative Breast Cancer
TSP domains: Thrombospondin domain
UTR: Untranslated region
